# Supplementary material for: Learning curve and surgical outcome of robotic assisted colorectal surgery with ERAS program
Source: Sci Rep. 2022 Nov 29;12:20566. doi: 10.1038/s41598-022-24665-w (PMC9709162; doi:10.1038/s41598-022-24665-w)
Supplement: Supplementary file 2 — Supplementary Information 2. [file 41598_2022_24665_MOESM2_ESM.zip › RAL Raw data-Ñ[▒K/093/Patho_ñw╝╨░O▒Kñσ.pdf]

臺中榮民總醫院 臺中市臺灣大道四段1650號 病理部活體組織切片 臨床醫學實驗及病理診斷中心 檢驗(查)報告

[redacted]

Pathologic diagnosis:
1. Sigmoid colon, robot anterior resection --- a. Status post polypectomy with chronic inflammation and submucosal fibrosis. Stitch and pigment deposition at the previous polypectomy site noted. No residual malignancy found. The bilateral cut ends are free of tumor. b. Metastatic adenocarcinoma, moderately differentiated (1/13), of mesocolic lymph nodes.
2. IMA root, dissection --- Unremarkable fibrovascular tissue and lymph nodes (0/2).
3. Colon, cut end, distal, resection --- Chronic inflammation.

Ancillary study for diagnosis:
Deep cut done on section C and D.

Prognostic and predictive factor:
(Combined with the previous specimen S1018275)
1. Tumor size: 0.3 cm (measured on slide).
2. Depth of invasion: Tumor invades to submucosa.
3. Histologic grade: G2: Moderately differentiated.
4. Angiolymphatic invasion: Present.
5. Perineural invasion: Not identified.
6. Tumor deposits (discontinuous extramural extension): Not identified.
7. Macroscopic tumor perforation: Not identified.
8. Surgical Margins: a) Proximal Margin: Uninvolved by invasive carcinoma. b) Distal Margin: Uninvolved by invasive carcinoma. c) Radial or Mesenteric Margin: Uninvolved by invasive carcinoma.
9. Lymph node status: Involved (1/15).
10. Extranodal involvement: Not identified.
11. Tumor regression grading S/P CCRT: No known presurgical therapy.
12. Type of polyp in which invasive carcinoma arose: Not identified.
13. Additional pathologic findings: Not identified.
14. Pathological TNM Stage: pT1N1a(According to the eighth edition, American Joint Committee on Cancer Staging Guidelines for Tumors).
15. TNM descriptors: Not applicable.

Gross description:
The specimen consists of 1) a segment of colon, 15 cm in length and 5 cm in proximal cut end circumference, 5.5 cm in distal circumference, and mesocolic soft tissue. There is a polypoid lesion, 0.5x0.5 cm, labeled with stitch, noted with 4 cm to the nearest cut end. On cross section, the tumor invades to the mesocolic soft tissue with mm to the radial margin. There are two smaller polypoid lesions, 0.5x0.3 cm and 0.2x0.2 cm, reespectively, noted. The both cut ends are free of tumor grossly. 2) IMA root, a piece of tan yellow soft tissue, 5.8x2.3x0.8 cm. 3) distal cut end, a piece of tan mucosa-covered soft tissue, 2.3x2x0.8 cm. Representative part for section: A) proximal cut end B) distal cut end C-E) all of stitch labeled lesion F-G) polyp H-J) specimen 2-3 K-M) lymph nodes.

Note:
This case has been peer reviewed by two doctors.
#T-59300_2 #M-09413_2 0 1105 000000
#T-C4560_2 #M-81406_2 2 1105 00000F

已電子簽章

敬告：本單僅供醫師參考使用，不具任何申請效力！！
請病患妥為保管，若不慎遺失需自負個人資料外洩責任！
